# Transaxillary Capsulorrhaphy with Reimplantation to Correct Bottoming-Out Deformity in Breast Mycobacterial Periprosthetic Infection: A Case Report with Literature Review

**DOI:** 10.1055/a-2119-3835

**Published:** 2023-12-01

**Authors:** Tsung-Chun Huang, Jian-Jr Lee, Kuo-Hui Yang, Chia-Huei Chou, Yu-Chen Chang

**Affiliations:** 1 Department of Plastic and Reconstructive Surgery, China Medical University Hospital, China Medical University, Taichung, Taiwan; 2 School of Medicine, China Medical University, Taichung City, Taiwan; 3 Virtue Cosmetic Surgery Clinic, Taichung, Taiwan; 4 Division of Infectious Diseases, Department of Internal Medicine, China Medical University Hospital, China Medical University, Taichung, Taiwan; 5 Department of Surgery, China Medical University Hospital, China Medical University, Taichung, Taiwan

**Keywords:** bottoming-out deformity, capsular flap, transaxillary capsulorrhaphy, mycobacterial infection

## Abstract

Augmentation mammoplasty is one of the most popular cosmetic surgeries, but there is a high reoperation rate (29.7%) commonly due to capsular contracture, implant malpositioning, infection, and unsatisfactory size. Although infection only accounts for 2% of cases, its management is very challenging, especially with nontuberculous mycobacteria (NTM) infection. Breast prosthetic NTM infection is a rare but is a disastrous condition with an incidence of approximately 0.013%. Immediate salvage reimplantation is usually not suggested, and most studies recommend a gap of 3 to 6 months after combination antibiotics therapy before reimplantation. However, delayed reimplantation often leads to great psychological stress and struggle between the doctor and patient. We present the case report of successful reimplantation in treating prosthetic NTM infections in a 28-year-old female. We discuss a novel technique “transaxillary capsulorrhaphy” to correct the bottoming-out deformity. One year after the combination of antibiotics and surgery, the follow-up computed tomography scan showed complete remission of NTM without recurrence. We discuss the surgical technique in detail. The 1-year follow-up assessment (photos and dynamic video) revealed good cosmesis and reliable correction using the new technique. This report is the first formal description and discussion of one-stage reimplantation following NTM infections. Transaxillary capsulorrhaphy allows for a successful salvage operation when an implant is displaced. This approach provides highly favorable result in eastern women undergoing revision augmentation mammoplasty. This study reflects level of evidence V, considering opinions of respected authorities based on clinical experience, descriptive studies, or reports of expert committees.

## Introduction

Augmentation mammoplasty is one of the most popular cosmetic surgeries globally. However, a high reoperation rate after the surgery (29.7%) was reported in a recent cohort study. 1 The most common reasons for reoperation include capsular contracture, malposition, infection, or unsatisfactory size. 2 3 Although the incidence of wound infection is only 2%, its management is extremely challenging, especially in the case of nontuberculous mycobacteria (NTM) infection. Delayed signs of mycobacterial infection further render the treatment with prosthetics difficult.

Endoscopic transaxillary augmentation mammoplasty is the most accepted surgical intervention in Asian women because the incisional scar is hidden. 4 In the cases of revision, most women prefer to be operated on from the previous incisional scar. This fervent demand of Asian women promotes the surgeon to undertake transaxillary capsulectomy with immediate reimplantation. 4 For this purpose, Hung described a feasible method to achieve good cosmetic results. 2

Among implant malpositioning deformities, *bottoming* - *out* is the most challenging distortion, which may be further complicated by the presence of a concomitant NTM infection, but there is no evidence of such occurrence. We present the first case of treating NTM infection in a one-stage procedure using the transaxillary approach and correcting breast implant bottoming-out deformity with a transaxillary capsular flap.

In the past, immediate salvage implantation for NTM infection has not been suggested and most articles recommend a gap of 3 to 6 months after combination therapy before going for reimplantation. 5 To the best of our knowledge, only one case of NTM infection has been reported in a breast cancer female after mastectomy with successful implant reimplantation. 6 Some patients insist on preserving the cosmesis despite the risk of immediate reimplantation, and there is great psychological stress incongruity between the doctor and patient. A cohort study 5 described that only one case with breast prosthetic NTM infection has been reported in Taiwan in the past 30 years. However, we believe that the incidence of NTM infections is extremely underestimated because we encountered at least two cases in our hospital over the past year.

In this case report, we present the case of a patient with bilateral NTM infections occurring at different times (early and delayed break out) after previous augmentation mammoplasty, along with a bigger right implant with bottoming-out deformity and severe scar contracture in the left breast. She requested a “smaller” size on the right side and augmentation on the left side. We describe a successful one-stage revision surgery in this patient. As stated before, the only acceptable approach was via the previous axillary scar.

## Case



**Video 1** The video illustrates the neopocket after capsular flap capsulorrhaphy. The capsular flap was fixed to the chest wall with eight Ethibond sutures equally distributed along the new inframammary fold.


**Video 2** The video shows the strong and effective suspension of the capsular flap. Soft breasts and a stable inframammary fold position were observed 1 year after the operation. The natural dynamic results in satisfactory cosmetic outcomes.


**Fig. 1:**
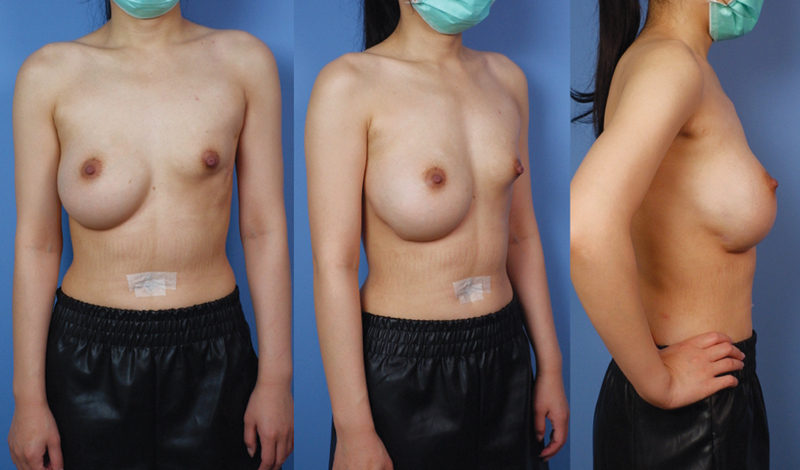
Images after treatment for left breast nontuberculous mycobacterial (NTM) infection revealed left breast scar contracture and deformity. The right breast had downward displacement and was “too big” as complained by the patient.

A 28-year-old otherwise healthy female presented with a 4-week history of erythema and painful skin ulceration of the left breast and chest wall. She had undergone two failed breast augmentation procedures (unsatisfactory cosmesis) performed at a private clinic in the past 7 months before visiting our hospital. On initial gross evaluation, there was swelling, tenderness, and erythematous skin probably due to infected fluid collection. Emergent removing of implant with partial capsulectomy and copious pocket irrigation with Betadine solution were performed, and the serous fluid and capsule were sent for culture. The specimen was negative for bacterial and fungal culture, but acid-fast stain (2 + ) and NTM culture revealed the presence of *Mycobacterium brisbanense* . The patient was prescribed the antibiotic amikacin for 1 week intravenously during admission, followed by oral doxycycline and ciprofloxacin for 8 months. The first episode of NTM infection of the left breast was now symptom-free and ready for reimplantation ( Fig. 1 ).

**Fig. 2:**
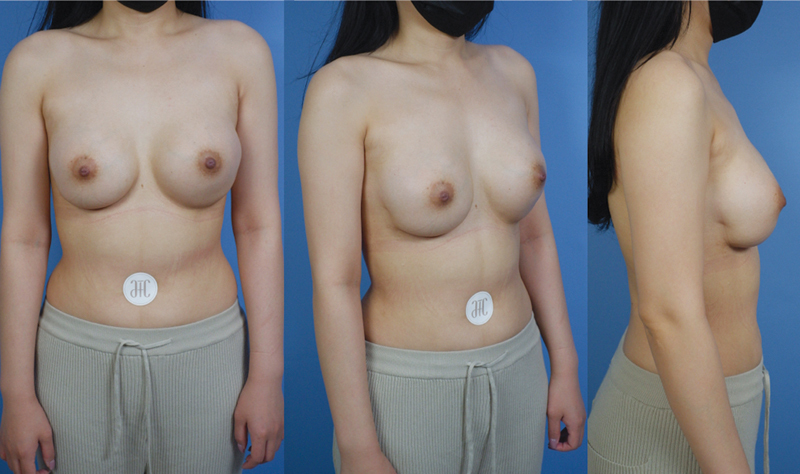
Postoperative results after 1 year. The downward displaced right breast and bilateral asymmetry were well corrected via bilateral transaxillary capsulorrhaphy. The desired right breast position and projection of direction can be achieved with reliable suspension.

After reimplantation, initial prophylactic antibiotic Augmentin was prescribed. However, an incidental new second infection with another NTM pathogen, *Mycobacterium fortuitum* complex, was diagnosed 2 weeks after bilateral revision mammoplasty with the implant. New implants were placed in the neopocket with the dual plane. The fluid collection from bilateral breasts tested negative for acid-fast stain—the mycobacterial culture report for the left breast revealed negative findings, but the newly reported NTM was detected 2 weeks after this reimplantation. Because bilateral breast suction drains were left in situ after the operation, the patient experienced a few episodes of low-grade fever before detecting the NTM. Other nonspecific symptoms included breast swelling, redness, and mild tenderness. Because she fervently refused to implant removal at this stage, she was admitted to receive combinational antibiotics (cefoxitin, levofloxacin, and amikacin) for the right breast NTM infection for 2 weeks. Suction drains for bilateral breasts were removed 1 month after reimplantation until the daily amount was < 10 mL. The fluid from the suction drain was regularly tested for NTM culture. The final fluid collection was sent to culture before removing drainage, which was negative after 1 month of treatment. The patient continued on combination antibiotic therapy with linezolid, doxycycline, and ciprofloxacin for another 6 months. The postoperative cosmetic result was to the patient's satisfaction. After 1 year of follow-up, there were no signs of NTM infection recurrence bilaterally and good cosmesis and symmetry were retained a year after the operation ( Fig. 2 ).

Different techniques were applied to bilateral breasts. The left breast presented with severe atrophy of the lower pole and diffuse scaring because of the two failed augmentations and NTM infection damage. During the transaxillary method, the subpectoral plane was performed initially in the axilla and upper pole area, and then, diffuse scaring at the lower and lateral breast was released until a tension-free neopocket was obtained. The dissection of the lower pole should be made along the deep plane along the deep fascia fiber of the external oblique and serratus muscle. Partial myotomy of the serratus muscle and medial-lower border pectoralis muscle can be made until releasing all the scar band tissue. The neopocket included the upper pole in the submuscular plane and the lower and lateral sides in the subfascial and subglandular planes. The surgeon must be cautious while releasing the scaring inside the pocket to keep enough thin tissue beneath the previous NTM infection scar. A SilkSurface implant, Motiva (Establishment Labs Holdings Inc., NY), DEMI 340 mL, was inserted.

**Fig. 3:**
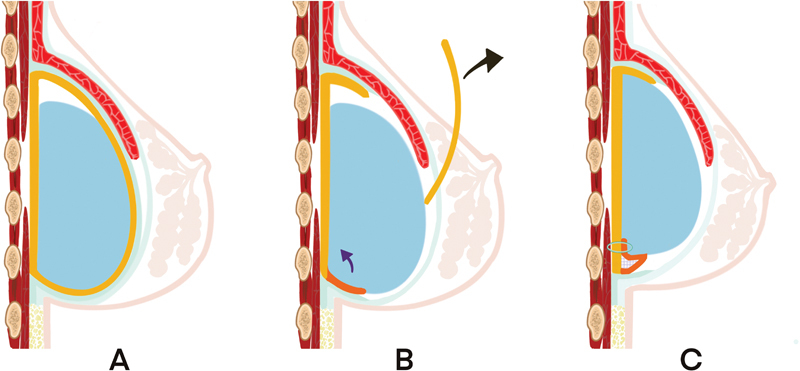
A detailed technique of the capsular flap suspension in right breast revision mammoplasty. ( **A** ) The downward displacement of the submuscular implant resulted in the wrong inframammary fold (IMF) position and inappropriate nipple projection. ( **B** ) After partial capsulectomy to remove the anterior capsule (yellow), a neopocket can be created along the pectoralis muscle and deep fascia. Too lower IMF can be corrected by capsular flap (orange) suspension. ( **C** ) After the suspension of the capsular flap was made, the rough surface between the flap and chest wall was electrocoagulated (gray spot) to enhance adhesion.

The right breast presented as a too big implant and developed a bottoming-out deformity with malpositioning and chronic seroma. Along the previous right axillary scar, the subpectoral dual plane was dissected, and the previous texture implant (400 mL) was removed. Approximately 60 mL of serous fluid was encountered after opening the capsule. Fluid was sent for bacterial, fungus, and mycobacterial culture. A partial capsulectomy was performed along the upper pole ( Fig. 3 ), and the lower pole and lateral pocket were evaluated in detail to design a new pocket with the same size as that of the implant and inframammary fold (IMF). The lower part of the previous capsule was harvested using endoscopic electrocoagulation equipment. A crescentic capsular flap, which contained soft tissue and the previous capsule and which was approximately 2 to 4 cm in width, was elevated from 3 to 9 o'clock. Next, endoscopic capsulorrhaphy was performed along the new higher designed IMF to correct the bottoming-out deformity. A suture material with 2-O Ethibond was applied in eight fixation points with equally distributed suspension tension from the capsule flap to the chest wall ( Video 1 ). Additionally, we performed electrical coagulation on the rough surface along the capsular flap where it contacted the chest wall to trigger inflammation and enhance the flap suspension. This neopocket can be seen as the submuscular dual plane. A same-sized SilkSurface Motiva implant DEMI 340 mL was inserted.

Before the bilateral breast implant was inserted, both pockets were massively irrigated with 3 L of normal saline solution. The implant was soaked with antibiotic solution and then inserted with the “no-touch” technique. 2 After reimplantation, a 15-Fr Jackson-Pratt drain was inserted in the bilateral implant pockets and the patient was asked to wear a compression garment around the breast for 3 months.

**Fig. 4:**
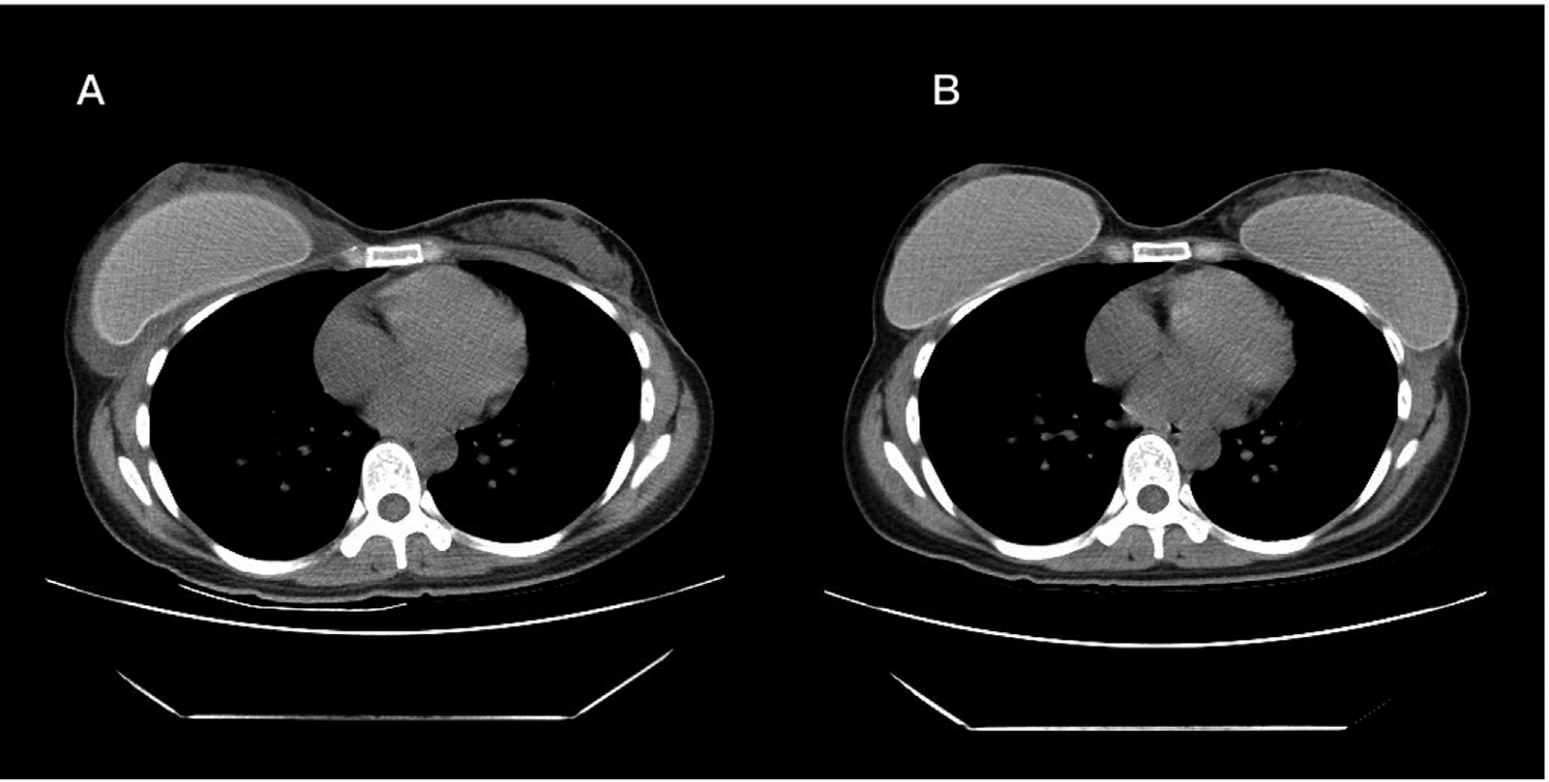
( **A** ) Computed tomography (CT) scan before revision mammoplasty showed a deflated left breast and implant fluid accumulation in the right breast, which was diagnosed as a nontuberculous mycobacterial (NTM) infection later. ( **B** ) Postoperative CT scan (after 1 year) showed no recurrent NTM infection in bilateral breast implant pockets.

One year after the one-staged revision mammoplasty via the transaxillary approach, the dynamic video ( Video 2 ) showed reliable suspension for the bottoming-out correction. The breast implants were soft without any downward displacement, had good cosmesis, and had symmetrical results. Six months after the discontinuation of the antibiotics, the postoperative 1 year photograph and computed tomography scan showed no recurrence of the NTM infection ( Fig. 4 ).

## Discussion

Breast periprosthetic infection occurs in approximately 1 to 2% of augmentations and 1 to 35% of breast reconstructions. Bacterial causes account for the majority of cosmetic failures and reconstructions. Although NTM is a very rare infection, the recent surge in aesthetic tourism has contributed to sporadic outbreaks in many countries. 5 The estimated incidence of NTM infections in breast prostheses was approximately 0.013% as per a 1983 survey. 7 The true incidence, however, remains unknown, and most researchers agree that the reported rates are probably underestimated. Therefore, early detection and awareness regarding NTM infections are important to treat and salvage the final results.

The hydrophobic nature of mycobacteria promotes the formation of a periprosthetic biofilm. 5 Earlier, salvage replantation in one stage was not recommended and the mainstay treatment included implant removal and delayed implantation (after 3–6 months). 3 5 In our patient, we applied the SilkSurface Motiva implant, which has a lesser rough surface to refill the pocket with less biofilm adhesion. 8 The suction drain was placed along the lower border of the implant until the daily amount was < 10 mL. Definitive removal of infected tissue and following antibiotics 4 to 6 weeks are thought to be successful to reduce biofilm formation. 9 Furthermore, intraoperative anterior capsulectomy, followed by postoperative intravenous combination antibiotics (amikacin, levofloxacin, and cefoxitin) from the second week to the fourth week, and 6 weeks of oral antibiotics (linezolid, doxycycline, and ciprofloxacin) significantly helped in controlling the NTM infection. This was evident from the 1-year follow-up that confirmed that there was no recurrence.

It is known that bilateral involvement is extremely rare in patients with NTM infection, accounting for only 8.3%. 3 The most perplexing factor in our case was that the prosthesis in the left breast developed acute NTM infection with *M. brisbanense* and that in the right breast had late infection with *M. fortuitum* complex. After 8 months of a complete course of antibiotics for the infection in the left breast (doxycycline and ciprofloxacin), the patient did not complain of any discomfort, except the right breast being “too big.” No signs of any infection were present, which made the preoperative diagnosis very challenging. Possible origin of these two different NTM pathogens comes from her previous two failure mammoplasties at a local clinic, which means contamination is not favorable.

The patient also asked for revision mammoplasty bilaterally, that is, a third-time augmentation of the left breast, a third revision of the right breast for a smaller implant, the correction of the right breast bottoming-out deformity, and an operation only via the previous transaxillary scar. We successfully changed to a smaller implant, that is, from Mentor Texture Moderate Plus 380 mL to Motive DEMI 340 mL, and contralateral augmentation with the same-sized Motiva DEMI 340 mL. Postoperative assessment at 1 year revealed symmetrical results without recurrence of bottoming-out. The patient remains satisfied with the cosmesis and natural dynamics.

Although salvage reimplantation is usually not recommended, approximately 12% of the patients received immediate explantation and reimplantation. 5 In such cases, the struggle between the immense psychological impact on the patient and the stress of achieving the desired outcome safely on the operating surgeon affects decision-making. Most successful salvage operations have been performed in bacterial infections, and there is only one case of an NTM infection salvaged in a one-stage breast cancer reconstruction. 6 Our patient is the first case salvaged for cosmetic purposes. Adherence to the use of a full course of intravenous and oral antibiotics, along with postoperative clear suction drainage, was the key factor ensuring surgical success in our case. Owing to the long incubation period of NTM infections, we used a postoperative prophylactic combination of cefoxitin, levofloxacin, and amikacin for 14 days and oral antibiotics for 8 months in our patient.

The correction of the bottoming-out deformity was mainly achieved via the nipple areolar 10 or the IMF approach. 11 12 13 14 Different capsular flaps 10 11 12 13 14 or acellular dermal matrix 15 capsulorrhaphy techniques have been described based on either the anterior or inferior capsule. However, although the majority of Asian women opt for the transaxillary approach when requiring a revision mammoplasty, there is no detailed description of this technique in the literature. The main disadvantage of the transaxillary approach is the long operative distance and questionable suture strength applied via the endoscope. To counter this, our technique comprises a relatively reproducible and reliable method, that is, by harvesting a vascularized flap with inferior-based blood supply, long-term suspension can be achieved. The soft and dynamic postoperative results ( Video 2 ) substantiate the reliability of the technique. A 2-cm wide soft tissue should be accompanied along the capsular flap to increase vascularity, and 6 to 8 sutures should be made to anchor to the chest wall along the crescentic flap. Additionally, the rough surface between the chest wall and flap induced an inflammatory change by electrocoagulation to enhance long-term strength. Therefore, using this capsular flap, implant malposition and bottoming-out deformity can be corrected safely via the transaxillary approach.

This report is the first formal description and discussion of one-stage reimplantation following NTM infections, combined with the correction of the bottoming-out deformity through transaxillary capsulorrhaphy. The 1-year follow-up further corroborated the reliability of the suspension technique and its safety while treating NTM infections.
